# iTRAQ-Based Proteomics Analyses of Sterile/Fertile Anthers from a Thermo-Sensitive Cytoplasmic Male-Sterile Wheat with *Aegilops kotschyi* Cytoplasm

**DOI:** 10.3390/ijms19051344

**Published:** 2018-05-02

**Authors:** Gaoming Zhang, Jiali Ye, Yulin Jia, Lingli Zhang, Xiyue Song

**Affiliations:** College of Agronomy, Northwest A&F University, Yangling 712100, China; zhanggaoming@nwafu.edu.cn (G.Z.); yejiali@nwafu.edu.cn (J.Y.); jiayulin@nwafu.edu.cn (Y.J.); zhanglingli@nwafu.edu.cn (L.Z.)

**Keywords:** anther proteomics, hybrid wheat, isobaric tags for relative and absolute quantification (iTRAQ), thermo-sensitive cytoplasmic male sterility

## Abstract

A “two-line hybrid system” was developed, previously based on thermo-sensitive cytoplasmic male sterility in *Aegilops kotschyi* (K-TCMS), which can be used in wheat breeding. The K-TCMS line exhibits complete male sterility and it can be used to produce hybrid wheat seeds during the normal wheat-growing season; it propagates via self-pollination at high temperatures. Isobaric tags for relative and absolute quantification-based quantitative proteome and bioinformatics analyses of the TCMS line KTM3315A were conducted under different fertility conditions to understand the mechanisms of fertility conversion in the pollen development stages. In total, 4639 proteins were identified, the differentially abundant proteins that increased/decreased in plants with differences in fertility were mainly involved with energy metabolism, starch and sucrose metabolism, phenylpropanoid biosynthesis, protein synthesis, translation, folding, and degradation. Compared with the sterile condition, many of the proteins that related to energy and phenylpropanoid metabolism increased during the anther development stage. Thus, we suggest that energy and phenylpropanoid metabolism pathways are important for fertility conversion in K-TCMS wheat. These findings provide valuable insights into the proteins involved with anther and pollen development, thereby, helping to further understand the mechanism of TCMS in wheat.

## 1. Introduction

Wheat is one of the most important food crops and large amounts of wheat are consumed each year [1]. Hybrid vigor or heterosis is a widespread phenomenon in many plants, and it is a very useful method for improving the yield and quality of crops such as sunflower, rice, rape, maize, and sorghum [2]. However, although previous studies have focused on the development of hybrid wheat, the scale of planted hybrid wheat is still very small due to the lack of an optimum system for hybrid production [3,4].

Previously, a “two-line hybrid system” was developed using thermo-sensitive cytoplasmic male sterility (TCMS) in *Aegilops kotschyi* (K-TCMS). KTM3315A, a K-TCMS wheat line, developed at Northwest A&F University, China, is characterized as a complete male sterile at low temperatures (daily average temperature <18 °C in Zadoks growth stages 41–59), but it is capable of producing self-pollinated seeds when the temperature is above 20 °C [5]. Moreover, due to its simple recovery and maintenance, as well as the lack of negative cytoplasmic effects, the sterility of KTM3315A can potentially be used widely in hybrid wheat breeding [6]. Thus, the two-line breeding system based on K-TCMS has great potential value in wheat production.

Proteomics is a critical complement to gene expression studies because proteins are the end products of genes and are more directly associated with cellular metabolism and biological processes. The scope of classical two-dimensional (2D) gel-based proteomic approaches is subject to several technical constraints, including the capacity for separation, discrimination effects, complex operation procedures, limited detection of low abundance and hydrophobic proteins, and low identification rate of proteins, which can make it difficult to obtain satisfactory results when analyzing over a large dynamic range [7,8]. In recent years, the proteome profiles of many cytoplasmic male sterile (CMS) plants have been studied in different development stages, thereby identifying numerous differentially abundant proteins (DAPs), including those related to ribosome subunits, proteasome subunits, energy metabolism (ATP synthesis subunits), protein synthesis and degradation, ribonucleoproteins, flavonoid synthesis, and transcription factor subunits; these proteins have been defined as contributors to male sterility in rice [9], cotton [10], cybrid pomelo [11], Welsh onion [12], cabbage [13], watermelon [14], and rapeseed [15]. However, a complete reference proteome is not available due to the complexity of the wheat genome and proteome, so obtaining the proteome profiles of CMS wheat remains difficult. Thus, given that few proteome profiles are available for CMS wheat, proteomic analysis can be used to complement transcriptional data and improve our understanding of male sterility. ITRAQ is a powerful technique that can enable the detection of more proteins than traditional 2D gel electrophoresis (2-DE) and multiplex quantitative comparison of fractionated samples [16,17]. The first study of the wheat proteome using iTRAQ investigated the response of wheat to drought stress [18]. However, the proteome profiles of TCMS wheat have not been investigated in great detail.

In this study, we investigated the proteome of wheat anthers using iTRAQ and identified the DAPs related to fertility conversion by analyzing the anther proteomes of KTM3315A in various developmental stages under different fertility conditions. The DAPs identified are representative of a variety of metabolic pathways such as oxidative phosphorylatin, phenylpropanoid biosynthesis, protein translation, synthesis, folding and degradation, and starch and sucrose metabolism. In order to determine their roles in anther development and fertility conversion in wheat, we investigated the potential effects of these proteins on male fertility and their possible biological functions. We identified some metabolic pathways related to wheat anthers and pollen development, thereby providing new insights into the mechanism of TCMS in wheat.

## 2. Results

### 2.1. Anther Development and Phenotypic Characterization

**Figure 1:**
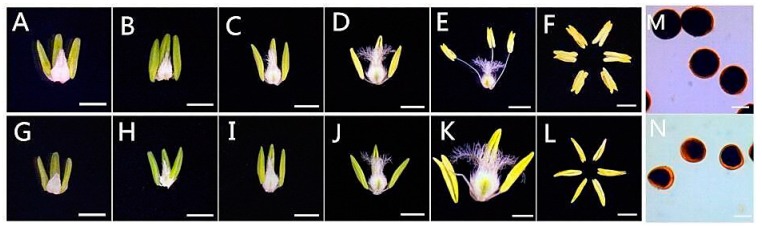
Comparison of plant anthers and I_2_–KI staining in sterile/fertile conditions (AF: (**A**–**F**,**M**); AS: (**G**–**L**,**N**)). (**A**,**G**) Tetrad stage; (**B**,**H**) early uninucleate stage; (**C**,**I**) later uninucleate stage; (**D**,**J**) binucleate stage; and (**E**,**F**,**K**,**L**) trinucleate stage. Scale bars are 500 μm in (**A**–**F**) and (**G**–**L**); 50 μm in (**M**,**N**).

Anthers were collected from KTM3315A under specific growth conditions and in various developmental stages. Compared with KTM3315A in the fertile condition (designated as AF), KTM3315A in the sterile condition (designated as AS) had normal anthers in the first few stages, but there were many differences in its phenotypic characteristics during the trinucleate stage (TNP) (Figure 1E,F,K,L). In contrast to AS, AF could produce numerous viable grains and they were completely stained by 2% I_2_–KI (Figure 1M,N). Compared with the empty, flat anthers of AS, the anthers of AF were round, greener, brighter, and normal sized. Thus, these results, and those obtained in our previous study, indicate that the fertility of KTM3315A plants can be changed by higher temperature and that they have stable thermo-sensitive traits [19].

**Figure 2:**
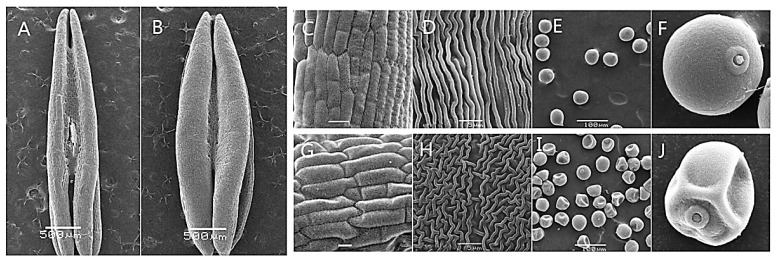
Comparison of scanning electron micrographs of AF (**B**–**F**) and AS (**A**,**G**–**J**). (**C**,**D**,**G**,**H**) outer epidermal ells; (**E**,**F**,**I**,**J**) trinucleate cells. Scale bars are 500 μm in (**A**,**B**); 100 μm in (**C**,**E**,**G**,**I**); 5 μm in (**D**,**H**); and 30 μm in (**F**,**J**).

In order to further understand male fertility in KTM3315A under different fertility conditions, we observed the outer epidermal surfaces of anthers with scanning electron microscopy during the TNP stage (Figure 2A–J). The fertile anther skin cells were significantly larger than the sterile anther cells (Figure 2C,D,G,H), although the fertile anthers were significantly plumper with a more rounded phenotype (Figure 2A,B), whereas, the sterile AS grains appeared to be deformed and shrunken (Figure 2E,F,I,J). These results demonstrate that higher temperature could change the fertility and anther morphology during anther development.

### 2.2. Assessment of Sequencing Results and Workflow Followed to Obtain Anther Proteomes

In order to further understand the molecular mechanism of fertility conversion in the KTM3315A wheat, we used fertile and sterile anthers in three development stages to quantitatively analyze the proteome with iTRAQ, where we identified the DAPs involved with fertility conversion during anther development. Global profiling of the quantitative proteome was performed using whole developing anthers from the uninucleate (UNP), binucleate (BNP), and trinucleate (TNP) stages. Using LC-MS/MS analysis of the iTRAQ labeled wheat anthers, 11,292 unique peptides representing 4639 proteins were detected. Of the detected proteins, 3155 (68%) were associated with at least two unique peptides. The median sequence coverage for the detected proteins was 25% (Supplementary Figure S1, and detailed information is provided in Supplementary Tables S1 and S2). The proteins detected were representative of a mass range from 10 to 100 kDa, of which the proteins in the range of 90–100 kDa represented only 2.6% (Supplementary Figure S2). The abundance levels of proteins were compared and analyzed in different samples, which showed that the protein abundance levels were generally identical. The data indicated consistent representation of the detected proteins in the selected samples, thus, facilitating the comparative analysis.

**Figure 3:**
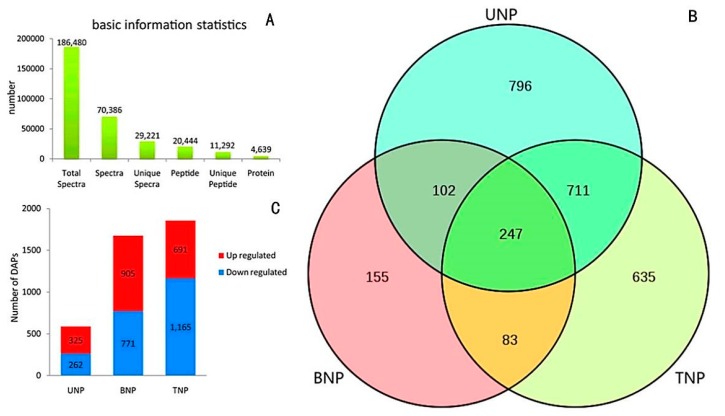
(**B**) Venn diagram; (**A**,**C**) accumulation histogram comparing the differentially abundant proteins (DAPs) in AS and AF. The numbers of DAPs are shown in the different stages. (**A**) Basic statistics; (**B**) Venn diagram of the DAPs in the three stages; (**C**) histogram showing the numbers of increased or decreased DAPs in the three stages (UNP, uninucleate stage; BNP, binucleate stage; and TNP, trinucleate stage).

We measured many DAPs in different samples in the three stages. According to the Venn diagram in Figure 3B, the AF/AS represent the total differential proteins of AF and AS at each stage, respectively. Some DAPs exist only at one stage; among the DAPs, we found that more DAPs were specifically abundant in the final two stages (635/796, BNP/TNP) than the first stage (155, UNP); just 247 DAPs were shared by all three stages (Figure 3B, and detailed information is provided in Supplementary Table S3). Moreover, by using an accumulation histogram (Figure 3C, and detailed information is provided in Supplementary Table S4) we can gain better insight into the magnitude of increase/decrease in DAPs during the three stages of anther development in AS and AF. At UNP, 1325 DAPs increased and 262 DAPs decreased. However, most of the DAPs (905) increased at BNP, and 771 decreased. Because UNP and BNP are the critical stages of anther development during fertility conversion, temperature changes lead to a lot of protein abundance changes, the number of proteins with an increased abundance is higher than the number proteins with a decreased abundance at UNP and BNP, the protein changes contribute to wheat fertility conversion and the plant’s response to temperature change. At TNP, the decreased protein (1165) is much higher than the increased protein (691), probably due to the anther developmental maturity at TNP.

### 2.3. Gene Ontology (GO) Enrichment Analyses of the DAPs

**Figure 4:**
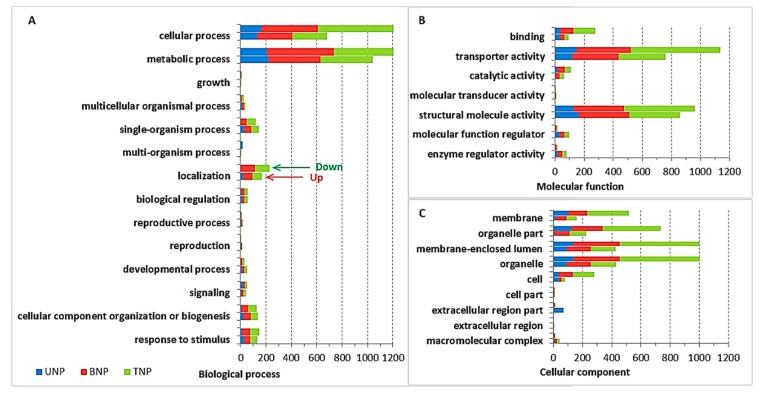
Gene Ontology (GO) analysis of the differentially abundant proteins (DAPs). (**A**) Biological process; (**B**) Molecular function; (**C**) Cellular component. The vertical axis shows the annotations of the GO classifications for DAPs. The horizontal axis represents the numbers of proteins annotated in each pathway, i.e., cellular components, molecular function, and biological process, and the proportion represents the number of proteins. UNP represents the uninucleate stage proteins, BNP represents the binucleate stage proteins, and TNP represents the trinucleate stage proteins. Red arrows indicate the decreased expression of DAPs and green arrows denote the increased expression of DAPs.

In the three stages, the enriched GO functions represent the different terms related to pollen development in AF and AS (Figure 4, and detailed information is provided in Supplementary Table S5). Among the biological process functions, the main DAPs were related to metabolic processes, cellular processes, localization, response to stimulus, and single-organism processes. Among the cellular components functions, the DAPs associated with cell parts, macromolecular complexes, membranes, and organelles differed from the total background proteins. Among the molecular functions, the DAPs associated with binding, catalytic activity, and structural molecule activity differed greatly from the background proteins. Thus, we suggest that normal pollen formation development was defective at low temperatures in AS, whereas high temperatures induced the enrichment of specific proteins, thereby leading to fertility conversion in the *Ae. kotschyi* K-TCMS wheat line.

### 2.4. Kyoto Encyclopedia of Genes and Genomes (KEGG) Enrichment Analysis of DAPs

**Figure 5:**
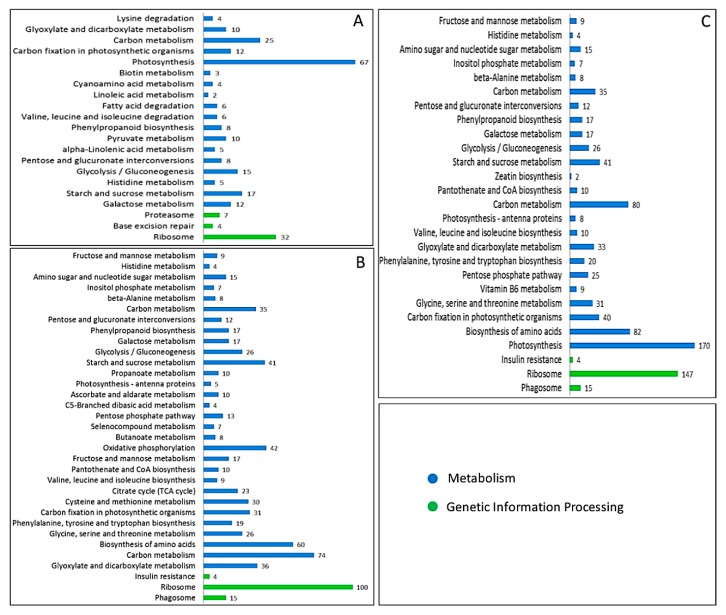
Main DAPs enriched in the top 20 pathways in UNP (**A**), BNP (**B**), and TNP (**C**).

We also made comparisons with the KEGG database in order to predict possibly important pathways. The results indicated the main DAPs enriched in the top 20 pathways in each stage (Figure 5, and detailed information is provided in Supplementary Table S6) mainly comprised ribosome, starch and sucrose metabolism, carbon metabolism, photosynthesis, biosynthesis of amino acids, phenylpropanoid biosynthesis, fructose and mannose metabolism, and carbon fixation in the photosynthetic organism. For the ribosome pathway, numerous DAPs were enriched in the three stages (100 in UNP, 101 in BNP, and 86 in TNP), where many of the DAPs that increased/decreased in each stage were associated with anther development and fertility conversion. The DAPs related to starch and sucrose metabolism were highly enriched and most of these DAPs were annotated in the first two stages. The pollen development process is a metabolic process that involves starch and sugar accumulation, so we suggest that the DAPs related to starch and sucrose metabolism are involved with pollen development and fertility conversion. DAPs related to carbon metabolism and photosynthesis were also enriched, particularly those involved with energy metabolism in plants, photosynthesis and carbon fixation, and cytoplasmic and mitochondrial energy metabolism related to carbon metabolism. There were differences in the protein abundance levels (increased/decreased) in the three stages in fertile and sterile plants, such as NADP-dependent malic enzyme, acetyl-coenzyme Asynthetase, glucose-6-phosphate 1-dehydrogenase, and fructose-1,6-bisphosphatase (Table 1). Thus, we suggest that there is a need for more energy during anther and pollen development in fertile plants, thereby facilitating normal anther development and fertility conversion. Many studies have also shown that starch and sucrose metabolism, energy metabolism and protein degradation, and synthesis pathways play important roles in male sterility in plants based on quantitative proteomics [10,11,20]. However, the molecular mechanisms that allow these pathways to affect plant fertility conversion have rarely been studied. In addition, many of the DAPs identified in KTM3315A may regulate fertility conversion and these DAPs can provide a deeper understanding of the mechanism of male sterility.

### 2.5. Analysis of Trends in DAPs

**Figure 6:**
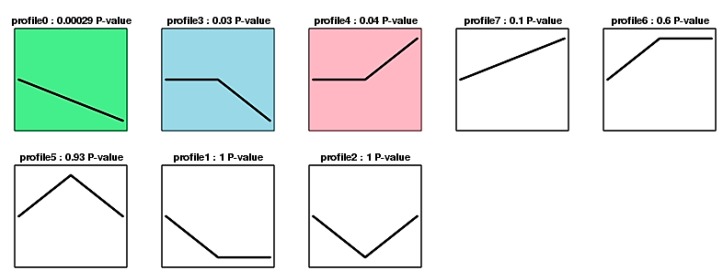
Cluster analysis of DAPs in AS and AF during different stages (UNP, BNP, and TNP). Based on fold changes of 1.2, the 127 DAPs could be divided into eight groups (profile 0 to 7) with correlated abundance patterns. The green represent profile 0; the blue represent profile 3; the red represent profile 4, were significantly different.

In this study, we detected 127 DAPs based on fold changes of 1.2 or <0.83 and a false discovery rate (FDR) of <0.05 as the selection criteria for the three stages. The results indicated that 127 DAPs could be divided into eight groups (from profiles 0 to 7) with correlated abundance patterns (Figure 6, and detailed information is provided in Supplementary Table S7). The profiles 0, 3, and 4 were significantly different, eighteen DAPs that grouped in profile 0 and 21 DAPs that grouped in profile 3 were greatly reduced in TNP; where these proteins were associated with carbohydrate metabolism, lipid metabolism, amino acid metabolism, and post-translational modifications, e.g., lipoxygenase, sucrose synthase 1, *S*-adenosylmethionine synthase, and calcium-dependent protein kinase SK5-like. By contrast, 30 DAPs in profile 4 were greatly increased in TNP. Interestingly, five were identified as defense proteins, six were associated with carbohydrate metabolism, two proteins comprised a pollen-specific protein and pollen allergen, two were related to flowering locus T, and the others were associated with protein synthesis and modification (Table 1). Thus, we suggest that the changes in these proteins were closely related to anther development and fertility conversion.

### 2.6. Relationships between DAPs and Their Corresponding Transcripts

**Figure 7:**
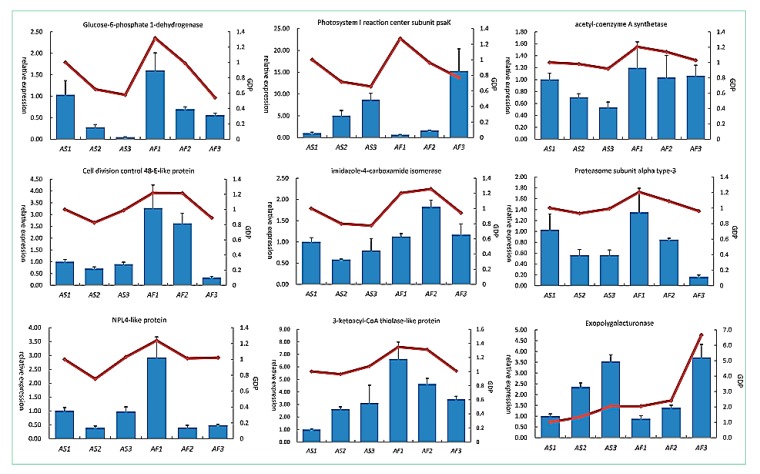
Expression levels of candidate DAPs according to qRT-PCR (histogram) and proteomics (line chart). qRT-PCR data represent the means based on three replicates and bars represent standard errors. GDP represents the level of expression of protein combined with proteomic data.

In order to evaluate the correlations between the mRNA and protein levels, we employed quantitative real-time PCR (qRT-PCR) to confirm the transcript levels of the DAPs, where nine DAPs were analyzed by qRT-PCR. RNA was extracted from sterile and fertile anthers in the three stages. As shown in Figure 6 (detailed information is provided in Supplementary Table S8), eight genes encoding 3-ketoacyl-CoA thiolase-like protein, exopolygalacturonase, NPL4-like protein, cell division control 48-E-like protein, acetyl-coenzyme A synthetase, glucose-6-phosphate 1-dehydrogenase, imidazole-4-carboxamide isomerase, and proteasome subunit alpha type-3 had similar mRNA and protein expression patterns in the three developmental stages. However, the gene encoding photosystem I reaction center subunit psaK had contradictory expression patterns compared with its protein expression patterns (Figure 7), indicating that its transcript levels were poor indicators of the corresponding proteins levels. These differences may be explained by the strict regulation of protein synthesis in multiple steps comprising transcription, translation, post-translational processing and modification, and finally the synthesis of the mature protein [21].

## 3. Discussion

In recent years, many studies have focused on heterosis in wheat, but no male sterile material is available for large scale breeding. Thus, K-TCMS is a special male sterile line where a change in temperature affects the fertility of pollen grains. Exploiting the advantages of KTM3315A may greatly shorten the breeding period to reduce the breeding process in a complex breeding program, facilitating the production of hybrid wheat. In the present study, we performed comprehensive iTRAQ-based quantitative proteome analyses of the anthers from KTM3315A during various developmental stages in different fertility conditions. We detected many DAPs in different fertility conditions and identified more DAPs than those detected in previous reports using traditional 2-DE and 2D-difference gel electrophoresis approaches [22,23]. The DAPs identified were mainly involved with translation and energy metabolism, protein synthesis and degradation, and defense/stress. Numerous DAPs increased in the anther development stages in AF, suggesting that most of these DAPs contribute directly or indirectly to normal anther development and fertility conversion in KTM3315A.

### 3.1. Starch and Sucrose Metabolism during Anther Development

Starch and sucrose metabolism provide the energy for all aspects of wheat growth and development. Sucrose and starch synthesis is regulated in a coordinated manner by many enzymes [24]. In the microspore development process, it is necessary to accumulate large amounts of nutrients to maintain normal development, such as starch and polysaccharides. These serve as important energy sources for the microspore, and are also important for anther development, where the starch and polysaccharide contents of pollen grains are closely related to fertility. The disordered metabolism of substances during anther development may lead to sterility [25], which can be detected by potassium iodide staining (Figure 1). Starch synthesis and accumulation are related to many enzymes (Table 2), including alpha-glucan phosphorylase and exopolygalacturonase, which activate the corresponding metabolic pathways to increase the synthesis and accumulation of starch. We found that alpha-glucan phosphorylase was increased during the TNP stage in the fertile line. Moreover, dextran phosphorylase affects starch and glycogen via the production of amylase. Thus, we suggest that the UNP and BNP stages are key stages for fertility transformation, where the increased expression of glucan phosphorylase contributes to the development of pollen grains as well as the synthesis and accumulation of starch and polysaccharides. By contrast, in the TNP stage, the pollen grains are nearly mature and the accumulation of starch and polysaccharides has finished, so the expression of glucan phosphorylase is lower. UTP-glucose-1-phosphate uridylyltransferase was increased in all three stages in the fertile line. UDP-glucose is the raw material used in the synthesis of various polysaccharides, and an increase in its content contributes to the formation of normal pollen grains. In addition, we found that the total soluble sugar content increased from UNP to TNP in the AS and AF, but the total soluble sugar content in the AF was significantly higher than the AS at the three stages (Supplementary Table S9). Previous studies showed that starch biosynthesis is regulated mainly by post-translational modifications of proteins, especially via phosphorylation [26]. The phosphorylation and dephosphorylation of proteins can modulate the activities of proteins. Many phosphorylases are also involved in the synthesis of starch and sucrose, such as hexokinase and glucose-1-phosphate adenylyltransferase. Thus, the phosphorylation sites may be important for the synthesis of starch and polysaccharides. Therefore, starch synthesis via phosphorylated or dephosphorylated DAPs may be important for fertility conversion in the TCMS wheat line KTM3315A. During the anther development stages, the number of DAPs involved with the metabolism of starch and sucrose was 24 in the UNP stage, 55 in the BNP stage, and 66 in the TNP stage. The changes in these proteins may affect the accumulation of starch and polysaccharides in pollen grains, which may partly explain why the microspore is empty in KTM3315A in the late development stage.

### 3.2. DAPs Related to Pollen Wall Formation

In a previous study, we found that the metabolism of phenylpropanoids and the formation of the tapetum, flavonoids, and phenolic compounds are very important for the formation of the cell wall in pollen grains during pollen development [27,28,29,30]. Phenylpropanoid metabolism produces many enzymes that regulate various metabolic pathways related to cell wall formation in pollen grains, such as those related to the biosynthesis of flavonoids and the biosynthesis of phenolic compounds. The pollen wall consists of four layers of cell structure, from inside to outside: tapetum, mesosphere, endothecium, and middle layer of epidermis. During pollen development, the tapetum cells surround the pollen, and secrete sporopollenin, which is the precursor of pollen, thereby affecting pollen wall formation of the final polymerization. Thus, it is extremely important to the development of normal mature pollen, which ensures the production of functional pollens grains. Flavonoids are the main materials required to synthesize pollen pigments. Many studies have shown that the conversion of phenylpropanoid compounds into flavonoids is crucial for normal anther development [31]. In the present study, we identified many DAPs related to phenylpropanoid metabolism, flavonoid metabolism, and proteins that regulate programmed cell death (PCD) by tapetal cells, e.g., 4-coumarate-CoA ligase and ABC transporter C. The expression of these DAPs increased in all three stages (Table 3). 4-Coumarate-CoA ligase is active in the phenylpropanoid pathway and it may contribute to the synthesis of flavonoids, which are the main materials used for the synthesis of pollen pigments, such as carotenoids. During anther development, the tapetal cells provide important materials used for pollen wall production via PCD [32,33]. A previous study found that calcium (Ca^+^) can cause PCD in the tapetum and lead to male sterility [10,34]. In the present study, we found that the expression levels of superoxide dismutase and catalase were increased in AS, whereas, calreticulin and calnexin-like protein were decreased in AF. Ca^+^ binds to calmodulin to activate calcium-dependent protein kinases in the tapetum, thereby leading to a cascade of reactions that regulate the expression of many genes to cause PCD in the tapetum. The upregulation or downregulation of DAPs may regulate degradation of the tapetum, which is related to fertility conversion in wheat.

### 3.3. DAPs Related to Disrupted Energy Metabolism during Anther Development

In the wheat growth process, the chemical reactions in many metabolic pathways produce energy, such as photosynthesis, glycolysis, oxidative phosphorylation, and the tricarboxylic acid cycle. These metabolic pathways satisfy the energy demands of plant growth. Previous studies have shown that the respiration rate in fertile anthers is higher than that in male sterile anthers, and the expression levels of enzymes related to energy metabolism are generally higher in fertile plants than those in male sterile plants [35,36], demonstrating that normal anther development requires more energy. In the present study, we identified many proteins related to energy metabolism using an iTRAQ-based quantitative proteomic approach, and we found that the expression levels of proteins involved with energy metabolism varied between the anthers of AS and AF plants in the anther development stages (Table 4). Many of the DAPs were involved with photosynthesis, glycolysis, oxidative phosphorylation, the tricarboxylic acid cycle, and oxidative phosphorylation of mitochondria in plant cells, where energy is released when substances are oxidized to enter the coupling reaction with ADP and inorganic phosphorus to synthesize ATP. Mitochondrial membrane ATP synthase (F_1_F_0_-ATP synthase) plays a crucial role in energy metabolism by converting ADP into ATP in the presence of a transmembrane proton gradient, and numerous energy metabolism proteins are involved with the tricarboxylic acid cycle [37,38]. The UNP stage is the critical period for fertility conversion in KTM3315A [19] and we found that many DAPs that related to energy increased/decreased during this period in the metabolic pathways mentioned above. The ATP synthetase subunits were identified, including ATPase subunit c and the ATP synthase CF1 epsilon subunit. These ATPase subunits interact with each other to regulate the activity of ATP synthase, and recent studies have shown that mitochondrial DNA encodes some ATP synthase subunits that are related to fertility [39]. One of the DAPs encodes a soluble inorganic pyrophosphatase-like protein, which acts as a phosphate and then acts on ADP to form ATP (Pi + ADP→ATP). These DAPs may affect the function of ATP synthase to alter cellular energy metabolism via the mitochondrial membrane, thereby affecting anther fertility.

In the present study, we found that the expression levels of many enzymes involved with glycolysis metabolism and the tricarboxylic acid cycle were increased/decreased in the fertile line compared with the sterile line, e.g., phosphofructokinase (PFK, increased enzyme), acetyl-CoA synthetase (increased enzyme), and fructose-1,6-bisphosphatase (decreased enzyme). PFK has a crucial regulatory role in glycolysis where it can convert fructose 6-phosphate and ATP into fructose 1,6-biophosphate and ADP to increase or decrease the rate of glycolysis in response to the cell’s energy requirements [40]. Fructose-1,6-bisphosphatase is an enzyme that converts fructose-1,6-bisphosphatase into fructose 6-phosphate in gluconeogenesis and the tricarboxylic acid cycle, which are both anabolic pathways, and these synthetic pathways cause the depletion of energy in the organism. In the CMS wheat line KTM3315A, the expression level of PFK increased and accelerated the pyruvate metabolism pathway to increase the synthesis of energy. In addition, fructose-1,6-diphosphate (FDP) is an important metabolic intermediate in the glycolytic pathway. FDP can act on the cell membrane and regulate enzyme activity in glucose metabolism, especially the activation of phosphofructokinase (PFK), resulting in increased ATP production. The expression level of fructose-1,6-bisphosphatase decreased and the glycolytic rate decreased, resulting in reduced anabolism and energy expenditure. Our findings indicate that these proteins play key roles in changes in fertility and the normal development of pollen grains, thereby providing insights into the variations in energy metabolism during the anther development stages.

### 3.4. DAPs Related to Protein Synthesis and Degradation

Anther development is a complex process that depends on the extensive regulation of protein synthesis and degradation. Protein synthesis is very important for normal wheat development, the formation of new cell proteins, protein degradation, and the output balance process. Proteins are synthesized via transcription, translation, post-translational processing, and modification, which finally lead to the synthesis of the mature protein, which is strictly regulated in multiple steps [21]. In this study, numerous DAPs were related to these biological processes. After gene transcription, mRNA precursors contain protein-coding exons and noncoding introns and require splicing by the spliceosome before a mature mRNA can be formed. The spliceosome comprises several protein subunits, and we found that nuclear cap-binding protein subunit 1 and UBP1-associated protein 2B were increased in the UNP stage, whereas serine/arginine-rich splicing factor 7 and serine/arginine-rich splicing factor RSZ23-like isoform X1 were decreased. In the BNP stage, other DAPs were increased or decreased, such as small nuclear ribonucleoprotein E, U6 snRNA-associated Sm-like protein LSm8, and U2 small nuclear ribonucleoprotein A. After the transcription and translation of a gene, the pre-protein needs to be folded in the endoplasmic reticulum to form a functional protein. Protein disulfide isomerases are involved with the formation of inter- or intramolecular disulphide bonds [41], and several protein disulfide isomerase genes have been cloned in wheat, where their transcriptional levels in endosperm cellularization demonstrate that they are associated with the deposition and synthesis of storage proteins [42]. Mitochondrial heat shock protein (Hsp) 60 and ismitochondrial chaperones have roles in multiple biological processes, including the degradation of unstable and misfolded proteins, transport of proteins between cellular compartments, and the folding and refolding of proteins [43]. In the present study, in the three pollen development stages, we identified some DAPs that directly or indirectly regulated the extension of peptide chains and protein synthesis, such as the heat shock cognate 70 kDa protein 4, Hsp70, cell division control 48-E-like protein, and NPL4-like protein (Table 5).

The main function of the proteasome is to degrade unneeded or misfolded proteins via proteolysis. In previous studies, 26S proteasome/ubiquitin proteins were identified by proteomic analyses in male sterile anthers [15,44], so we suggest that the proteasome may play a key role in anther development. The structure of the 26S proteasome comprises two subunits which interact to degrade unneeded and damaged proteins [45]. Many studies have shown that the 26S proteasome is involved with the regulation of numerous metabolic pathways, and thus it plays an important role in the regulation of proteins. According to our iTRAQ data, several proteasome subunits increased/decreased in abundance in KTM3315A. In the UNP stage, two proteasome subunits (proteasome subunit alpha type-7-A and proteasome subunit alpha type-3) decreased in abundance in KTM3315A. In the BNP stage, 12 proteasome subunits increased/decreased in abundance, including proteasome regulatory subunit N3, proteasome regulatory subunit N8, proteasome regulatory subunit T2, and proteasome subunit beta 2. These results suggest that proteasome activities might be defective in KTM3315A in some anther development stages and in fertility conversion. Any proteasome subunit defect is expected to lead to the accumulation of damaged proteins, reduced degradation rates for regulatory proteins, and dramatic decreases in the efficiency of signal transduction processes. Moreover, a feedback regulation mechanism exists between the activity of the 26S proteasome and the total protein content [46]. Thus, defects in the proteasome subunits in KTM3315A probably disrupted the balance of the 26S proteasome feedback regulation mechanism.

Analyses of plant proteasome mutants have confirmed that optimal proteasome activity levels are required for plant development, including male fertility and anther development, such as in *Arabidopsis* [47]. Thus, a rpt2a (26S proteasome AAA-ATPase subunit RPT2a) mutant exhibits pollen development failure and delayed flowering, while a rpt2a and rpt2b (26S proteasome AAA-ATPase subunit RPT2b) double mutant is defective in terms of the development of both the female and male gametophyte [48,49]. In the present study, we found that proteasome subunits rpt2a and rpt3a were decreased in AF plants during the BNP stage (Table 5), indicating that changes in the activities of these proteasome components may contribute to the normal development of pollen grains, and thus, to the successful production of functional pollen grains. According to these results and previous research [49,50], we suggest that changes in proteasome activity levels and protein degradation feedback loops may change the fertility of male sterile lines and transform them into fertile lines, which might be partially correlated with normal stamen and pollen development and fertility conversion.

## 4. Materials and Methods

### 4.1. Plant Materials, Plant Growth, and Anther Collection

KTM3315A, a K-TCMS wheat line, was used as the test material. In October 2015, the KTM3315A line was planted in flower pots at the experimental station of Northwest A & F University (108° E, 34°15′ N), China, under natural conditions in the field. On 2 April 2016, all of the pots were moved into two light incubators until the pollen grain production period (15 pots/each), where they had a light:dark regime of 14:10 h, and the temperature was set to 17 °C/15 °C for the sterile conditions and 22 °C/20 °C for the fertile conditions. These high and low temperatures were set for the AF and AS lines, respectively. The pollen developmental stages were identified during anthesis and we collected wheat anthers in the same developmental period as the materials used from AF and AS for proteome sequencing. Anthers in the UNP, BNP, and TNP stages were collected in separate tubes and stored in liquid nitrogen every other day, before storing at −80 °C until extracting the proteins and mRNA.

### 4.2. Anther Development and Phenotypic Characterization

Spikes and anthers were collected from AS and AF in different developmental stages. The anthers from the spikes in various development stages in different fertility conditions were observed under a light microscope. The I_2_–KI staining method was used to observe the mature pollen grains. The morphology of the anthers was observed in different periods and photographed under a Motic K400 dissecting microscope (Preiser Scientific, Louisville, KY, USA). The anthers in different developmental stages were fixed in formalin-aceto-alcohol as described by Zhang et al. [51], before observing the outer epidermis of the microspore. The morphology of the microspore in the TNP stage was examined by scanning electron microscopy using a JSM-6360LV system (JEOL, Tokyo, Japan).

### 4.3. Sample Preparation, Protein Extraction and iTRAQ Labeling

Anthers in the same development stage were collected from 100 AS and AF spikes for proteomic analysis. Total protein was extracted using the cold acetone method. Samples were ground to powder in liquid nitrogen, then dissolved in 2 mL lysis buffer (8 M Urea, 2% SDS, 1× Protease Inhibitor Cocktail (Roche Ltd., Basel, Switzerland)), followed by sonication on ice for 30 min and centrifugation at 13,000 rpm for 30 min at 4 °C. The supernatant was transferred to a fresh tube. For each sample, proteins were precipitated with ice-cold acetone at −20 °C overnight. The precipitations were cleaned with acetone three times and re-dissolved in 8 M Urea by sonication on ice. Protein quality was examined with SDS-PAGE. BCA protein assay was used to determine the protein concentration of the supernatant. 100 μg protein per condition was transferred into a new tube and adjusted to a final volume of 100 μL with 8 M Urea. 11 μL of 1MDTT (dl-Dithiothreitol) was added and samples which were incubated at 37 °C for 1 h. Then 120 μL of 55 mM iodoacetamide was added to the sample and incubated for 20 min, protected from light at room temperature. For each sample, proteins were precipitated with ice-cold acetone, and then re-dissolved in 100 μL TEAB. Proteins were then tryptic digested with sequence-grade modified trypsin (Promega, Madison, WI, USA) at 37 °C overnight. The resultant peptide mixture was labeled with iTRAQ. The labeled samples were combined and dried in vacuum.

Protein samples (100 mg each) from the anthers of AS and AF were digested with 50 mM trypsin. The digest of each sample was labeled using iTRAQ 8-plex kits (AB Sciex Inc, Foster City, CA, USA) according to the manufacturer’s instructions. AS samples from the UNP, BNP, and TNP stages were labeled with 111, 112, and 113 tags, respectively, and the AF samples from the UNP, BNP, and TNP were labeled with 114, 115, and 116 tags, respectively.

### 4.4. LC-MS/MS Measurements and Data Analysis

After iTRAQ labeling, the sample fractionation was performed before LC-MS/MS analysis by SCX chromatography using a Waters 600E HPLC system (Thermo Fisher DINOEX, Waltham, MA, USA). The iTRAQ labeled peptide mixtures were mixed and dried then reconstituted with 100 μL buffer A [25 mM NaH_2_PO_4_ in 25% acetonitrile (ACN), pH 2.7] and loaded onto a 4.6 × 250 mm Ultremex SCX column containing 5-μm particles (Thermo Fisher DINOEX, Waltham, MA, USA). The peptides were eluted at a flow rate of 1 mL/min with a gradient of buffer A for 10 min, 5–60% buffer B (25 mM NaH_2_PO_4_, 1 M KCl in 25% ACN, pH 2.7) for 27 min, 60–100% buffer B for 1 min. The system was then maintained at 100% buffer B for 1 min before equilibrating with buffer A for 10 min prior to the next injection. Elution was monitored by measuring the absorbance at 214 nm and fractions were collected every 1 min. The eluted peptides were pooled into 12 fractions, desalted with a ZipTip C18 column (Waters Corporation, Woburn, MA, USA) and vacuum-dried. Each fraction was resuspended in buffer A [5% ACN, 0.1% formic acid (FA)] and centrifuged at 12,000× *g* for 10 min, the final concentration of peptide was about 0.5 μg/μL on average. Ten microliters of peptide was loaded on a Eksigent nanoLC System (AB SCIEX, Framingham, MA, USA) by the autosampler onto a C18 trap column (5 μm, 100 μm × 20 mm). The peptides were separating using a self-packed analytical C18 column (3 μm, 75 µm × 150 mm). The samples were loaded at 5 μL/min for 10 min, then the 78 min gradient was run at 300 nL/min starting from 2 to 35% B (95% ACN, 0.1% FA), followed by a 5 min linear gradient to 60%, then, followed by a 2 min linear gradient to 80%, and maintained at 80% B for 4 min, and finally returned to 5% in 1 min.

Data acquisition was performed with a TripleTOF 5600+ System (AB SCIEX, Framingham, MA, USA) fitted with a Nanospray III source and a pulled quartz tip as the emitter (New Objectives, Woburn, MA, USA). Data was acquired using an ion spray voltage of 2.5 kV, curtain gas of 35 psi, nebulizer gas of 10 psi, and an interface heater temperature of 150 °C. The MS was operated in sensitive mode for TOF MS scans. For information dependent acquisition (IDA), survey scans were acquired in 250 ms and as many as 30 product ion scans were collected if they exceeded a threshold of 120 counts per second (cps) with a 2+ to 5+ charge-state. Total cycle time was fixed to 3.3 s. The Q2 transmission window was 100 Da for 100%. Four time bins were summed for each scan at a pulser frequency value of 11 kHz through monitoring of the 40 GHz multichannel TDC detector with a four-anode channel detect ion. A sweeping collision energy setting of 35 ± 5 eV coupled with iTRAQ adjust rolling collision energy was applied to all precursor ions for collision-induced dissociation. Precursor ions were excluded from reselection for 15 s (1/2 of average peak width). Raw data files acquired from the TripleTOF were converted into MGF files using Proteome Discoverer 1.2 and the MGF files were searched. Proteins were identified and quantified using Mascot software (version 2.3.02, Matrix Science Inc., Boston, MA, USA). The search parameters were as follows: peptide tolerance = 10 ppm and fragment mass tolerance = 0.05 Da; threshold set-off = 0.05 in the ion-score cut-off; tryptic peptides with >1 missed cleavage site; pyrophosphorylation of glutamine, variable oxidation of methionine, and iTRAQ labeling of tyrosine were set as variable modifications; and carbamido methylation of cysteine, iTRAQ labeling of lysine, and N-terminal amino group of peptides were set as fixed modifications. iTRAQ 8-plex was employed for simultaneous quantification during the search process. The search results were passed through additional filters before exporting the data. The filters were set as follows for protein identification: significance threshold *p* < 0.05 (with 95% confidence) and ion score or expected cutoff < 0.05 (with 95% confidence). The filters were set as follows for protein quantification: “median” for protein ratio type, minimum precursor charge = 2 and unique spectrum = 2, normalization by median intensities and outliers were removed automatically. The peptide threshold was set as described above for identification. Searches were performed against the coding sequence (CDS) protein database for wheat (Ensembl version 30, 100,344 proteins). Relative abundance was used to identify significant DEPs together with a *p*-value < 0.05.

### 4.5. Functional Category and Clustering Analyses of DAPs

Functional category analysis was performed with Blast2GO (Available online: http://www.gene-ontology.org) and Clusters of Orthologous Groups (COG) of proteins (Available online: http://www.ncbi.nlm.nih.gov/COG/). The protein ratios of DAPs were subjected to cluster analysis using the MultiExperiment Viewer application (version 4.8.1, Pittsburgh, PA, USA). K-Means clustering analysis with a Euclidean distance metric was used for cluster analysis. In comparisons with the wheat protein database, all of the proteins detected in this study were BLASTed to identify the closest match with a wheat *E*-value ≤ 10^−10^.

### 4.6. RNA Extraction and qRT-PCR Analysis

RNA was extracted from approximately 0.2 g samples of anthers (UNP, BNP, and TNP) from fertile and sterile plants. The primers used for qRT-PCR were designed with Primer Primer 5.0 (Primer, Palo Alto, CA, USA), verified by Primer-BLAST, and synthesized by Sangon Biotech (Shanghai) Co., Ltd., Shanghai, China (Supplemental Table S2). The primer pairs used for qRT-PCR are given in Supplemental Table S10. qRT-PCR was performed with a QuantStudioTM Real-Time PCR System (Applied Biosystems, Cambridge, MA, USA) using 2 RealStar Green Power Mixture (GenStar BioSolutions (Beijing) Co., Ltd., Beijing, China) with the following cycling parameters: 95 °C for 30 s, followed by 40 cycles at 95 °C for 15 s and 60 °C for 30 s. Each reaction mixture comprised of 20 μL containing 2 μL diluted cDNA and 1 μL of each primer, 10 μL of 2× RealStar Green Power Mixture, 0.5 μL ROX Reference Dye II (50×), and 6.5 μL RNase-free H_2_O. All of the qRT-PCR analyses were performed at least in triplicate. Relative gene expression levels were calculated using the 2^−ΔΔ^*^C^*^t^ method [52].

## 5. Conclusions

The K-TCMS wheat line, KTM3315A, is male sterile at low temperature (daily temperature <18 °C), but is capable of producing self-pollinated seeds when the temperature exceeds 20 °C during the pollen development stages. The K-TCMS-dependent two-line breeding system has great potential for future applications in wheat breeding. Thus, elucidation of the molecular mechanisms responsible for fertility conversion is very important. We employed iTRAQ-based quantitative proteomics analysis to investigate the proteins related to the change in fertility in K-TCMS wheat. Our results suggest that the failure of anther and pollen development in KTM3315A is associated with changes in proteins under the control of the nuclear genome or those related to mitochondrial functions, such as starch and sucrose metabolism, energy production, phenylpropanoid and flavonoid biosynthesis, and protein synthesis and degradation. In addition, our analysis of TCMS in the wheat line KTM3315A is highly significant for the use of heterosis in wheat.

## Figures and Tables

**Table 1:** Significantly enriched differentially abundant proteins in the three stages.

Gene ID	Description	BLASTp Result		AS vs. AF ^a^		
		*E*-Value	Score	UNP	BNP	TNP
*Traes_7DS_529BAB150.1*	Sucrose synthase 1	6.00 × 10^−8^	57	1.33	1.49	0.82
*Traes_6BL_FBF9DA7CE.1*	S-adenosylmethionine synthase 1	1.00 × 10^−96^	296	0.73	0.59	0.38
*Traes_5DS_69B96465C.1*	Calcium-dependent protein kinase	0	539	1.46	1.25	0.73
*Traes_7AS_EBD5F1F54.1*	Flowering locus T	6.00 × 10^−6^	46.2	1.96	2.17	3.06
*Traes_7BS_581AA844D.1*	Flowering locus T	3.00 × 10^−6^	46.6	1.96	2.17	3.06
*Traes_1DS_486DB7BFE.1*	NADP-dependent malic enzyme	0	555	1.21	1.39	1.76
*Traes_2AL_7A5A988CA.1*	Acetyl-coenzyme A synthetase	0	736	1.20	1.16	1.12
*Traes_2DL_06E6A2543.1*	Glucose-6-phosphate 1-dehydrogenase	7.00 × 10^−178^	520	1.32	1.52	0.94
*Traes_4AS_91D4C5213.1*	Fructose-1,6-bisphosphatase	2.00 × 10^−93^	288	0.73	0.72	0.64

^a^ Show the ratio of abundant proteins in fertile and sterile lines (the same as below).

**Table 2:** Differentially abundant proteins related to starch and sucrose metabolism in fertile and sterile plants.

GeneID	Description	BLASTp Result		AS vs. AF		
		*E*-Value	Score	UNP	BNP	TNP
*Traes_4DS_29F298799.1*	Glyco_hydro_1 domain-containing protein	1.00 × 10^−152^	449	−1.41	−3.52	−0.57
*Traes_3AL_9A3B8E4D9.1*	Alpha-glucan phosphorylase, H isozyme	8.00 × 10^−104^	329	2.20	2.10	1.57
*Traes_1AL_A1B2A8EB0.1*	ADP-glucose pyrophosphorylase large subunit	1.00 × 10^−99^	313	2.11	1.55	5.71
*Traes_6BS_0AFE47E4B.1*	UTP–glucose-1-phosphate uridylyltransferase	2.00 × 10^−140^	414	−1.07	−2.03	−1.08
*TRAES3BF066900020CFD_t1*	Hexokinase-6	6.00 × 10^−75^	250	−0.76	−1.30	−0.81

**Table 3:** Differentially abundant proteins related to pollen wall formation in fertile and sterile plants.

GeneID	Description	BLASTp Result		AS vs. AF		
		*E*-Value	Score	UNP	BNP	TNP
*Traes_6DL_7960654CF.2*	3-Ketoacyl-CoA thiolase-like protein	2.00 × 10^−52^	186	1.35	1.36	0.94
*Traes_2AL_43CCA70E9.1*	4-Coumarate-CoA ligase-like protein 9	3.00 × 10^−87^	285	2.75	2.87	0.61
*Traes_5DL_7D83C2AB2.1*	Calreticulin	6.00 × 10^−39^	145	−0.72	−1.54	0.07
*Traes_5BL_D71543428.1*	ABC transporter C family member 4-like	0	1107	0.83	0.57	1.15
*Traes_2BL_E8D5E0972.1*	3-Oxoacyl-synthase I, chloroplastic-like	1.00 × 10^−16^	83.6	1.35	−0.36	1.36
*Traes_6BS_D30CEBBC9.1*	Calnexin-like protein	4.00 × 10^−78^	259	−0.94	−2.50	−1.02

**Table 4:** Differentially abundant proteins related to energy metabolism in fertile and sterile plants.

GeneID	Description	BLASTp Result		AS vs. AF		
		*E*-Value	Score	UNP	BNP	TNP
*Traes_1AL_4F3CAE982.2*	Vacuolar proton-ATPase C subunit	2.00 × 10^−81^	257	2.00	2.22	1.92
*Traes_5DS_978062D3E.1*	ATP synthase CF1 epsilon subunit	1.00 × 10^−5^	44.3	−1.50	−1.37	−1.47
*Traes_6DS_27D11E4B8.2*		1.00 × 10^−5^	44.3	−1.50	−1.37	−1.47
*Traes_5BL_D12151D1D.1*	6-Phosphofructokinase 5	4.00 × 10^−11^	66.6	1.35	1.70	2.44
*Traes_5DL_27372150C.2*		2.00 × 10^−11^	67.8	1.35	1.70	2.44
*Traes_4AS_91D4C5213.1*	Fructose-1,6-bisphosphatase	2.00 × 10^−93^	288	−1.73	−1.78	−2.43
*Traes_4BL_B25B3CE481.1*		5.00 × 10^−93^	287	−1.73	−1.78	−2.43

**Table 5:** Differentially abundant proteins related to protein synthesis and degradation in fertile and sterile plants.

GeneID	Description	BLASTp Result		AS vs. AF		
		*E*-Value	Score	UNP	BNP	TNP
*Traes_1AL_3F7B8E94D.1*	Imidazole-4-carboxamide isomerase	4.00 × 10^−81^	423	1.20	1.57	1.22
*Traes_2AL_F73AFACB4.1*	UBP1-associated protein 2B-like	4.00 × 10^−22^	100	1.14	2.07	0.12
*Traes_6BL_0043F2EE5.1*	Serine/arginine-rich splicing factor 7	2.00 × 10^−15^	73.6	−1.80	−0.83	−0.01
*Traes_6AL_913A14974.2*	Serine/arginine-rich splicing factor	3.00 × 10^−16^	74.7	−1.80	−0.83	−0.01
*Traes_7BL_AEF6873D9.1*	Small nuclear ribonucleoprotein E-like	2.00 × 10^−6^	43.5	0.80	2.50	1.69
*Traes_1DL_E9432F054.1*	U6 snRNA-associated Sm-like protein LSm8	3.00 × 10^−10^	54.7	0.47	1.06	0.08
*Traes_5BL_21270EDFB.1*	U2 small nuclear ribonucleoprotein A′	7.00 × 10^−6^	48.1	−0.50	−1.09	−0.83
*Traes_1AL_51CED3DBF.1*	Heat shock cognate 70 kDa protein 4	0	872	−1.80	−1.14	−1.59
*Traes_4AS_B978C93FA.1*	Heat shock cognate 70 kDa protein	0	980	−1.25	−2.20	−1.51
*Traes_5DS_AC5D29D23.1*	Heat shock protein 83	0	556	−1.05	−2.13	−2.61
*Traes_3AL_75B505500.1*	DnaJ homolog subfamily C member 3	8.00 × 10^−18^	85.5	−0.71	1.50	0.10
*Traes_7AS_8B382E1D0.1*	Proteasome subunit alpha type-7-A	2.00 × 10^−74^	229	1.97	1.08	1.01
*Traes_5AL_2FB884126.2*	Proteasome subunit alpha type-3	3.00 × 10^−32^	121	1.03	0.85	−0.17
*Traes_2DL_C6C4CFB34.1*	26S proteasome non-ATPase regulatory subunit 7	3.00 × 10^−115^	341	−0.49	−2.00	−2.20
*Traes_2BL_2B983AD37.1*	26S proteasome regulatory subunit T3	0	697	−0.75	−1.25	−0.17
*Traes_4AS_6285AE7F6.1*	26S proteasome regulatory subunit T2	4.00 × 10^−133^	395	−0.55	−1.66	−0.08
*Traes_1BS_548536A26.2*	Proteasome subunit beta type-7-B	1.00 × 10^−89^	273	−0.23	−1.05	−1.87
*Traes_1BL_E29880146.1*	Cell division control 48-E-like protein	9.00 × 10^−97^	313	1.20	1.57	1.22
*Traes_6AS_E60FDC38F1.2*	NPL4-like protein	3.00 × 10^−11^	66.6	1.24	1.34	0.99

## Supplementary Materials

Supplementary materials can be found at http://www.mdpi.com/1422-0067/19/5/1344/s1.
