# Supplementary figures and images for: iTRAQ-Based Proteomics Analyses of Sterile/Fertile Anthers from a Thermo-Sensitive Cytoplasmic Male-Sterile Wheat with Aegilops kotschyi Cytoplasm
Source: Int J Mol Sci. 2018 May 2;19(5):1344. doi: 10.3390/ijms19051344 (PMC5983606; doi:10.3390/ijms19051344)

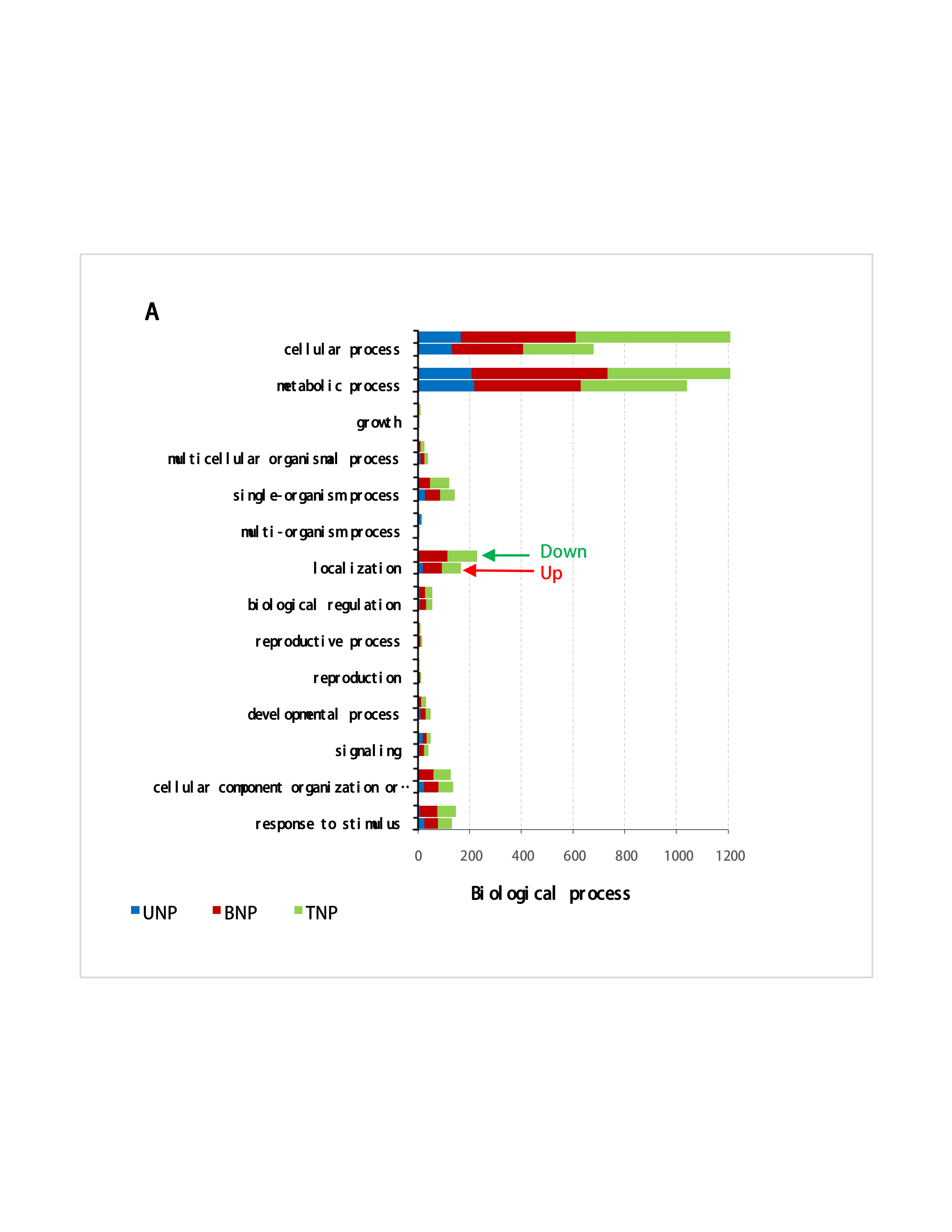

Supplement: Supplementary file 1 [file ijms-19-01344-s001.zip › Figures/Figure 4A.tif]

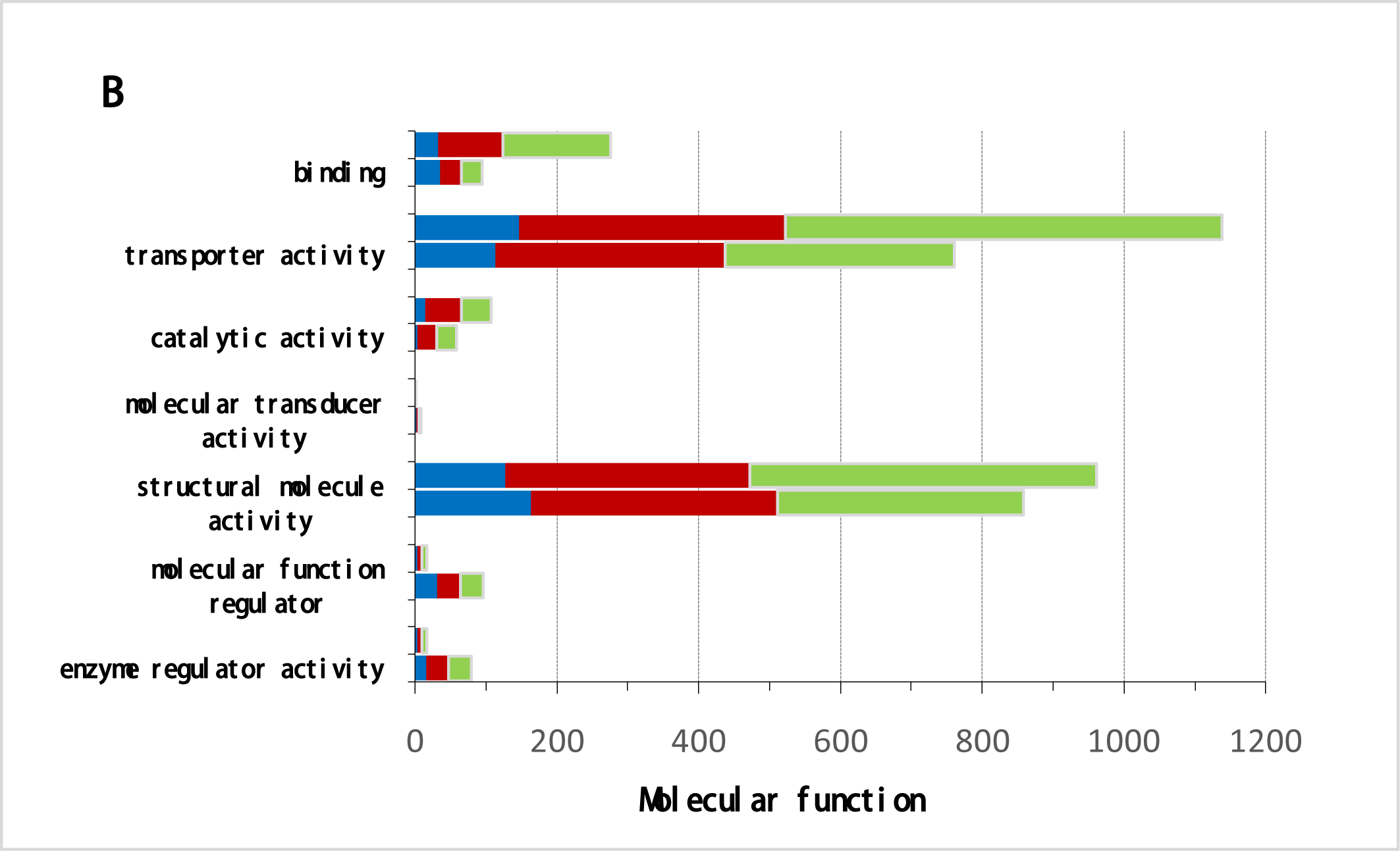

Supplement: Supplementary file 1 [file ijms-19-01344-s001.zip › Figures/Figure 4B.tif]

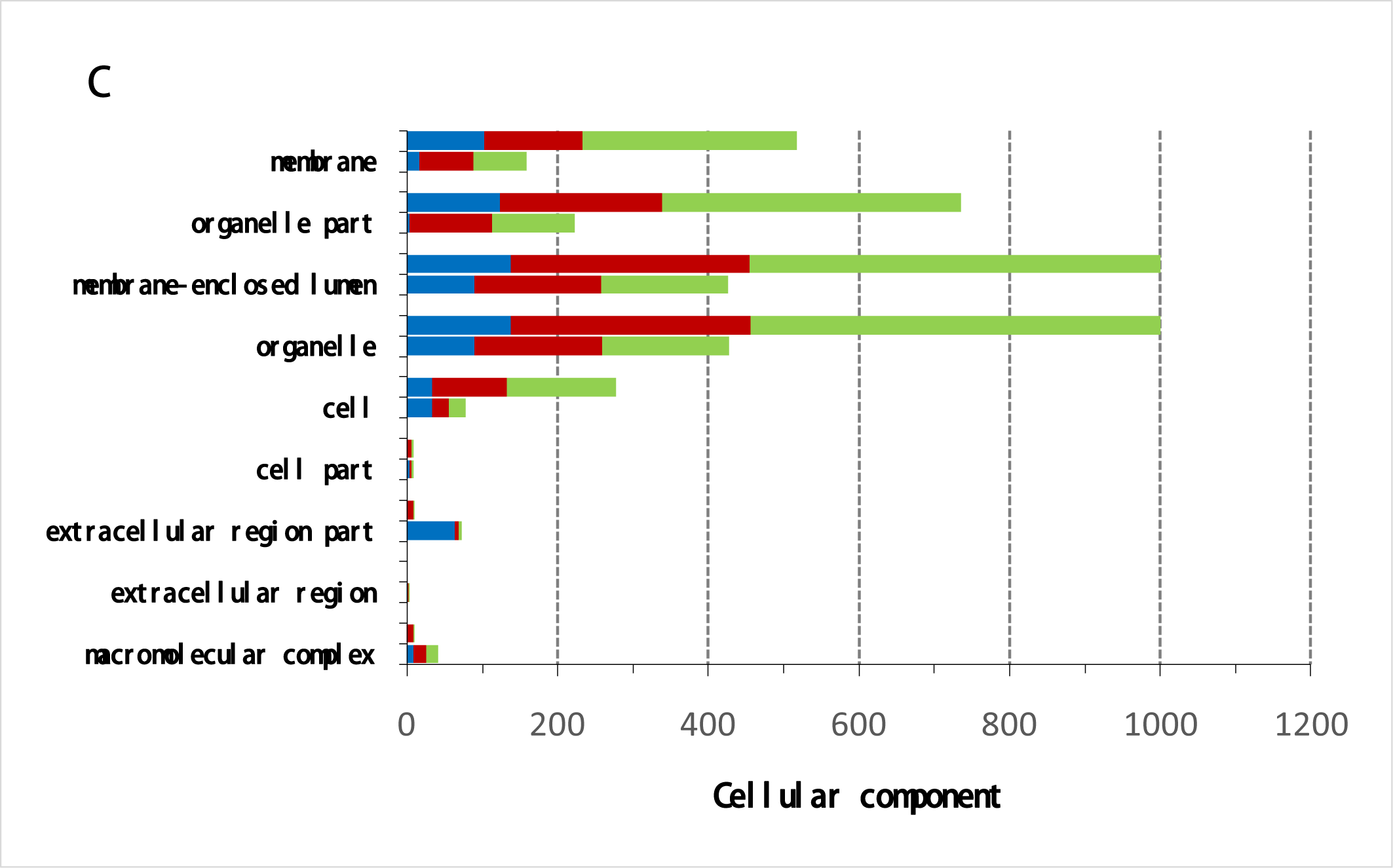

Supplement: Supplementary file 1 [file ijms-19-01344-s001.zip › Figures/Figure 4C.tif]

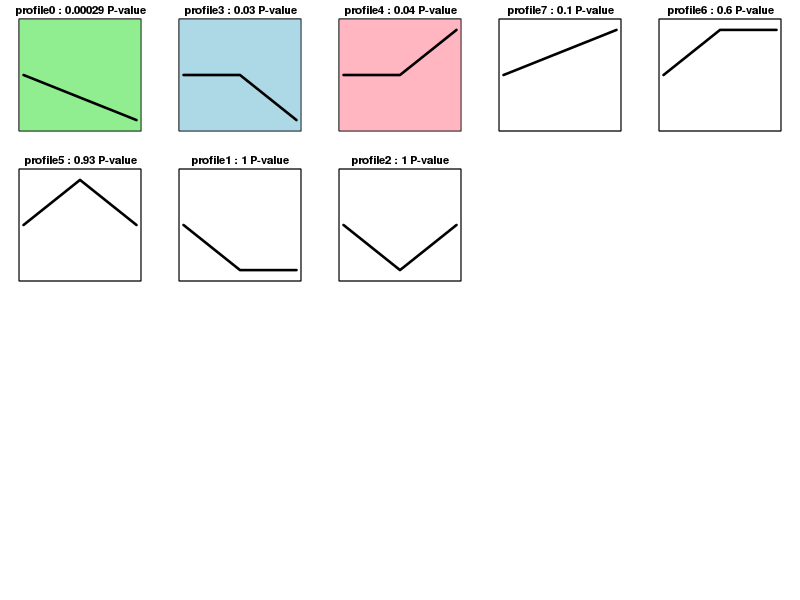

Supplement: Supplementary file 1 [file ijms-19-01344-s001.zip › Figures/Figure 6.tif]

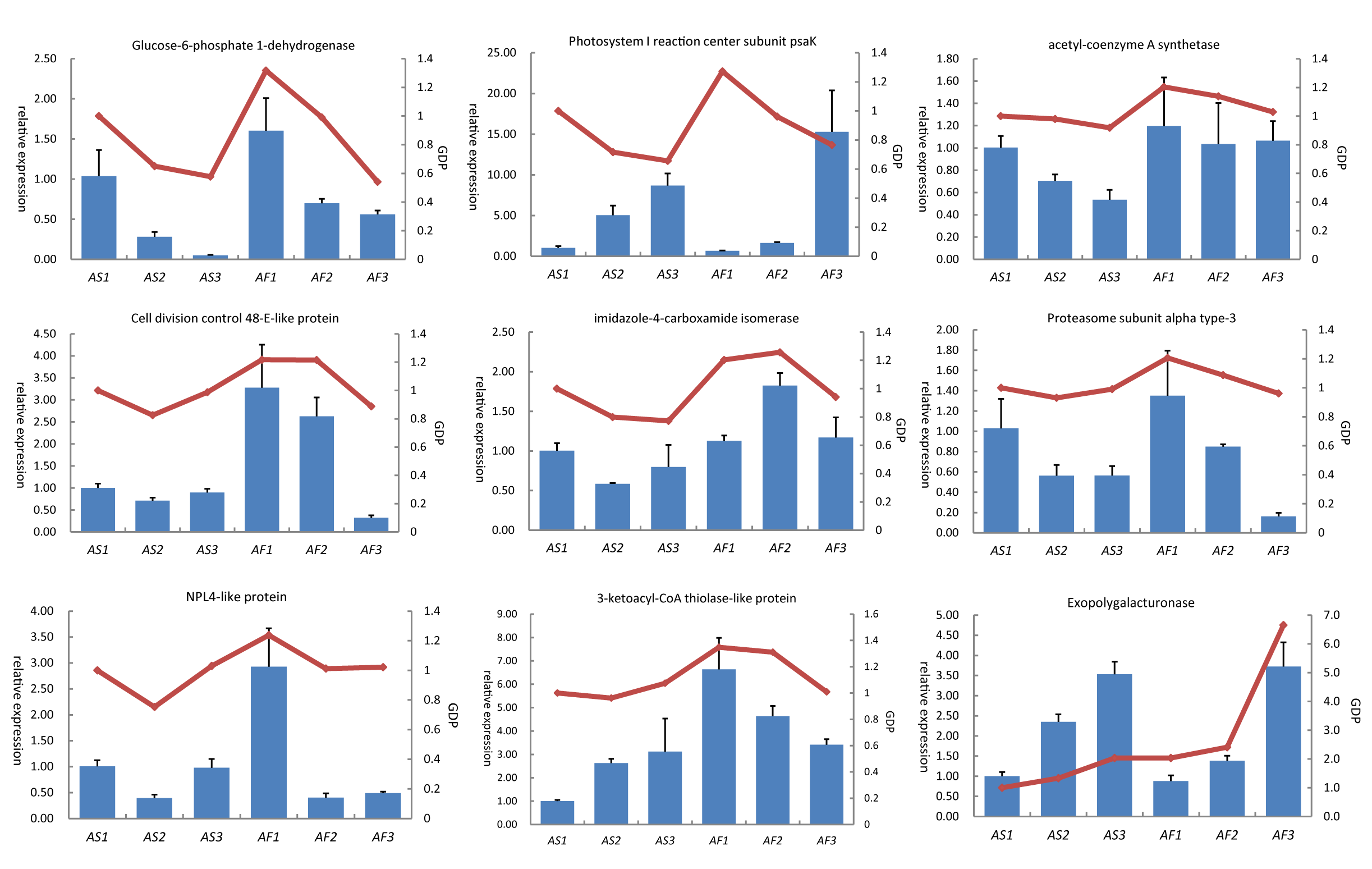

Supplement: Supplementary file 1 [file ijms-19-01344-s001.zip › Figures/Figure 7.tif]

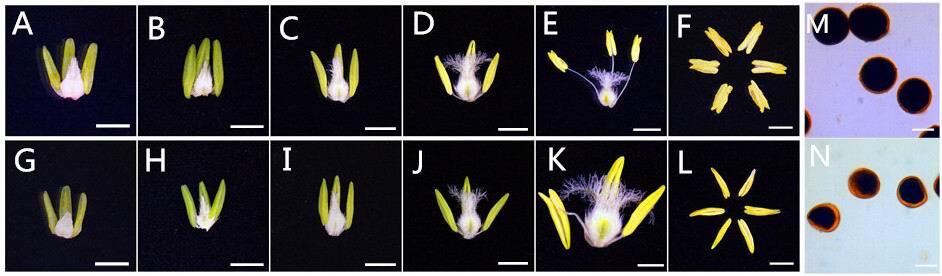

Supplement: Supplementary file 1 [file ijms-19-01344-s001.zip › Figures/Figure1.jpg]

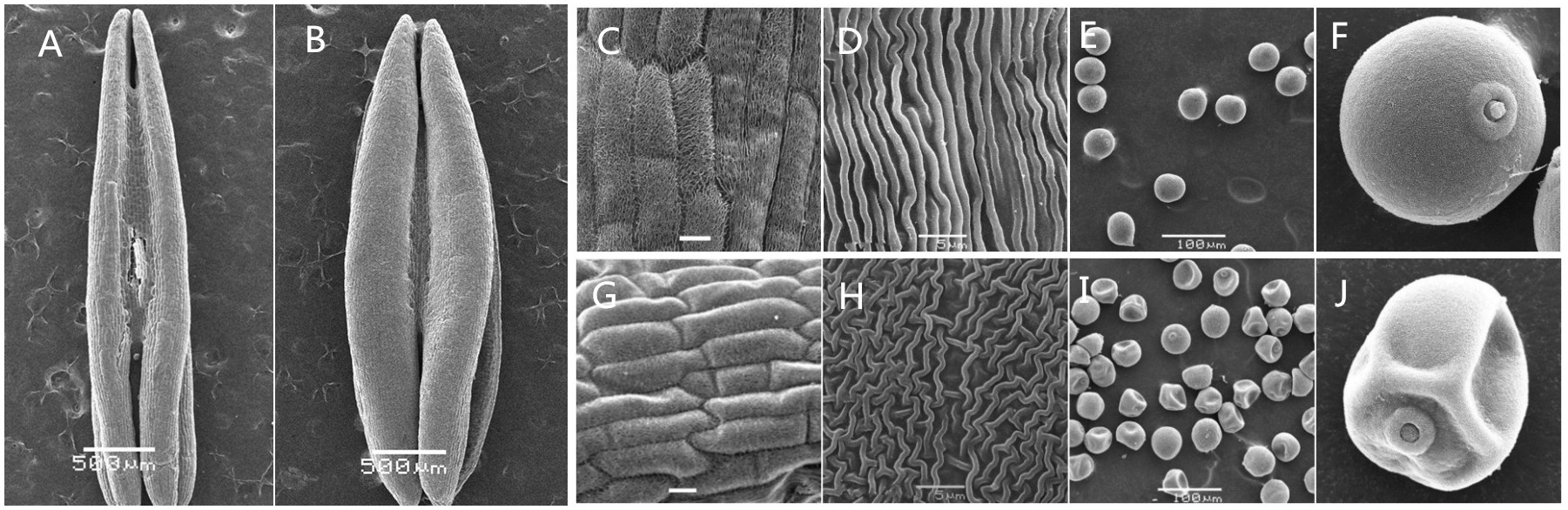

Supplement: Supplementary file 1 [file ijms-19-01344-s001.zip › Figures/Figure2.tif]

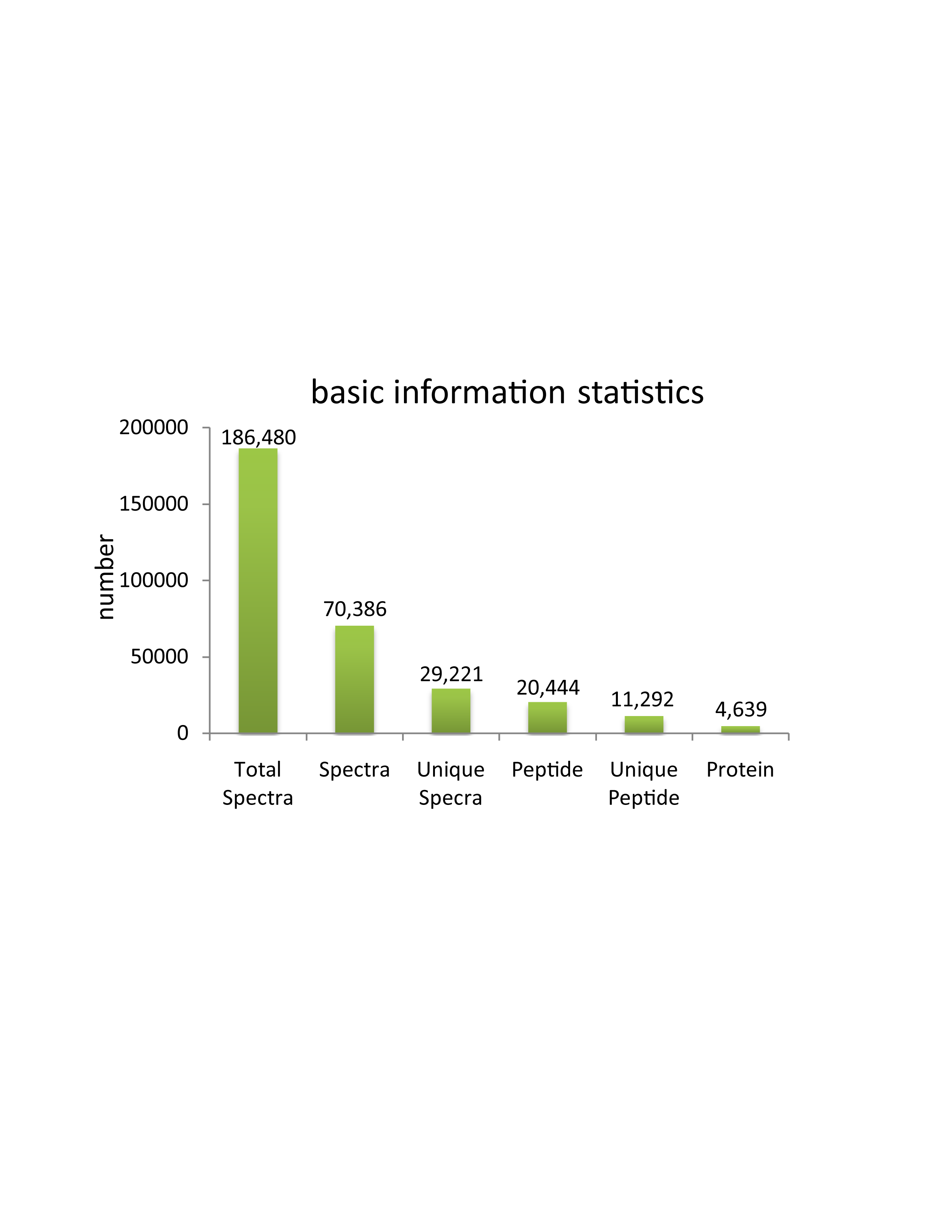

Supplement: Supplementary file 1 [file ijms-19-01344-s001.zip › Figures/Figure3A.tif]

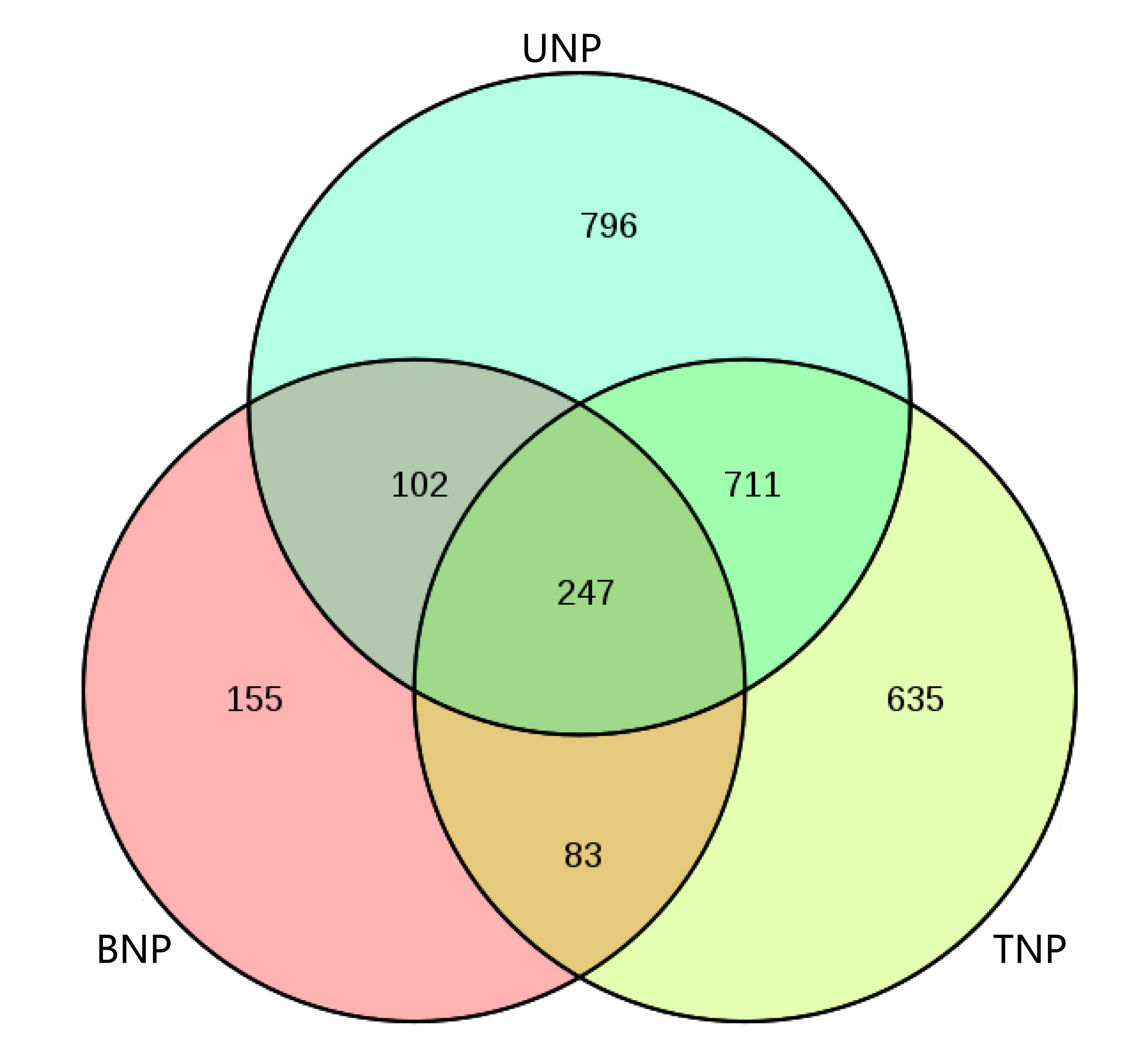

Supplement: Supplementary file 1 [file ijms-19-01344-s001.zip › Figures/Figure3B.tif]

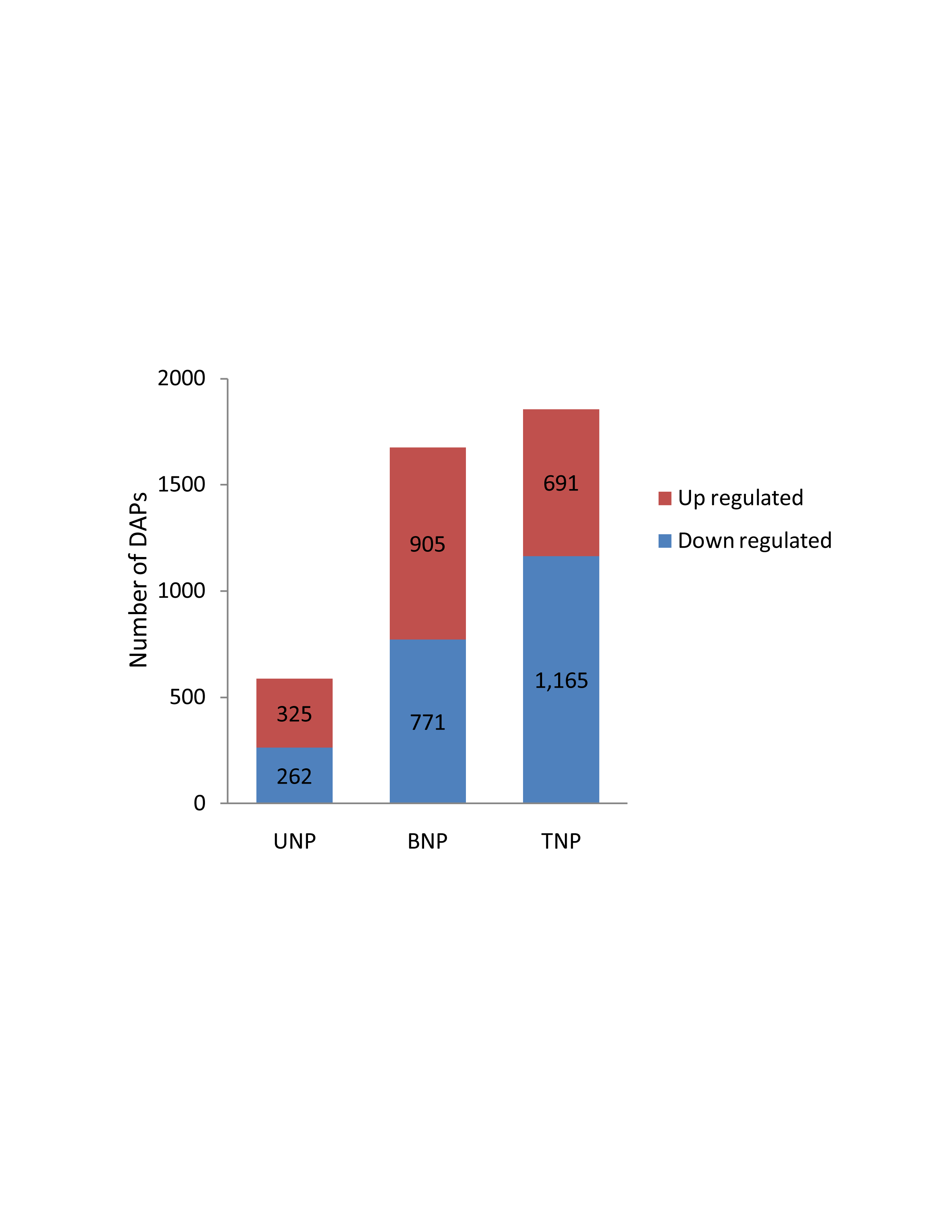

Supplement: Supplementary file 1 [file ijms-19-01344-s001.zip › Figures/Figure3C.tif]

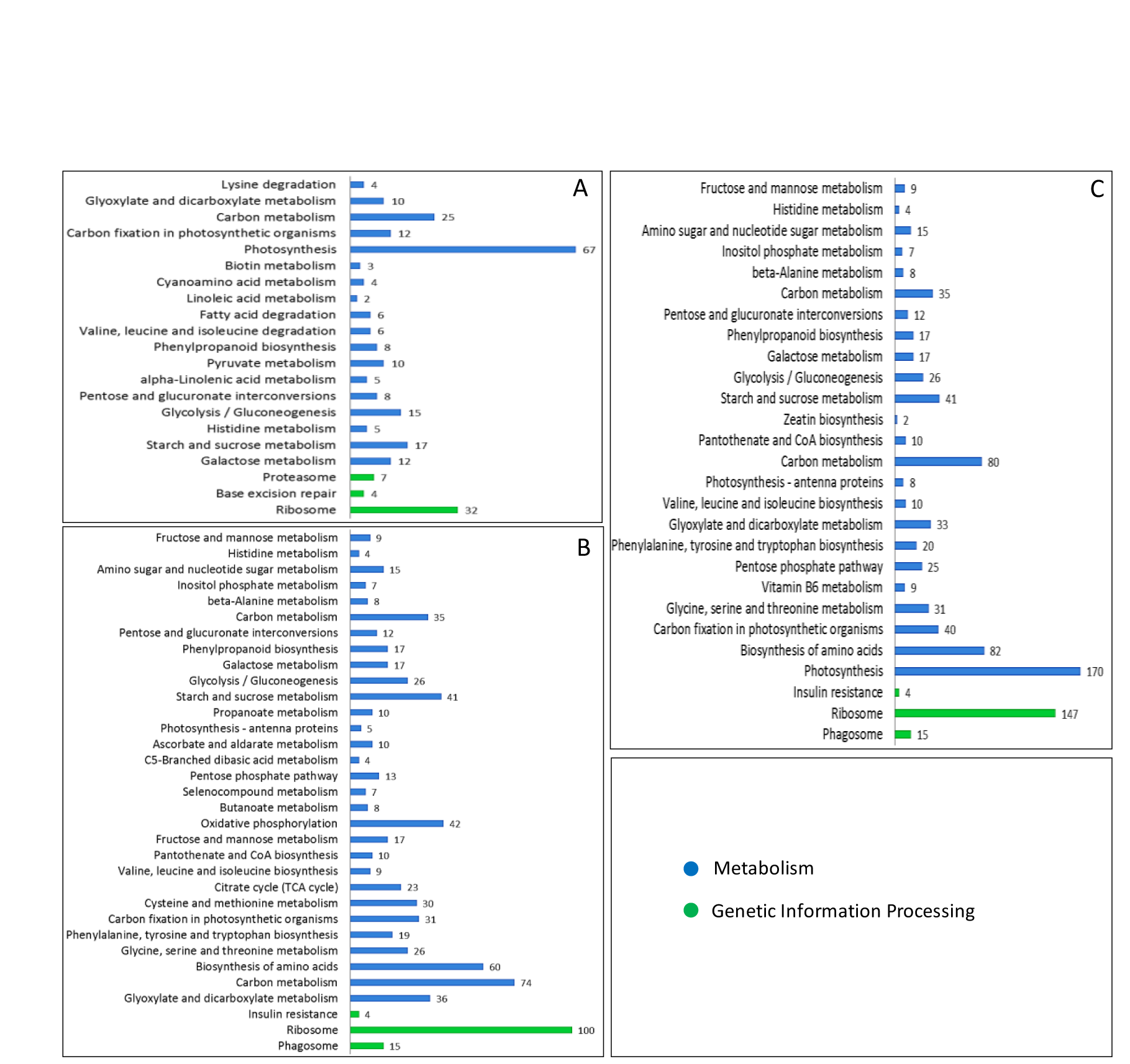

Supplement: Supplementary file 1 [file ijms-19-01344-s001.zip › Figures/Figure5.tif]
